# The potential negative impact of antibiotic pack on antibiotic stewardship in primary care in Switzerland: a modelling study

**DOI:** 10.1186/s13756-020-00724-7

**Published:** 2020-05-08

**Authors:** Julia Füri, Andreas Widmer, Delia Bornand, Christoph Berger, Benedikt Huttner, Julia Anna Bielicki

**Affiliations:** 1 Division of Infectious Diseases and Hospital Epidemiology, University Hospital Basel, Petersgraben 4, 4031 Basel, Switzerland; 2 Paediatric Pharmacology Group, University of Basel Children’s Hospital, Basel, Switzerland; 3 SwissNoso, National Centre for Infection Prevention, Bern, Switzerland; 4 Hospital Pharmacy, University Hospital Basel, Basel, Switzerland; 5 Division of Infectious Diseases and Hospital Epidemiology, University Children’s Hospital Zurich, Zurich, Switzerland; 6 Children’s Research Center, University Children’s Hospital Zurich, Zurich, Switzerland; 7 Infection Control Program and Division of Infectious Diseases, World Health Organization Collaborating Centre on Patient Safety, Geneva University Hospitals, Geneva, Switzerland; 8 Faculty of Medicine, University of Geneva, Geneva, Switzerland

**Keywords:** Antibiotic stewardship, Community prescribing, Children, Adults, Prescribing guidelines

## Abstract

**Background:**

In Switzerland, oral antibiotics are dispensed in packs rather than by exact pill-count. We investigated whether available packs support compliance with recommended primary care treatment regimens for common infections in children and adults.

**Methods:**

Hospital-based guidelines for oral community -based treatment of acute otitis media, sinusitis, tonsillopharyngitis, community-acquired pneumonia and afebrile urinary tract infection were identified in 2017 in an iterative process by contacting hospital pharmacists and infectious diseases specialists. Furthermore, newly available national guidelines published in 2019 were reviewed. Available pack sizes for recommended solid, dispersible and liquid antibiotic formulations were retrieved from the Swiss pharmaceutical register and compared with recommended regimens to determine optimal (no leftovers) and adequate (optimal +/− one dose) matches.

**Results:**

A large variety of recommended regimens were identified. For adults, optimal and adequate packs were available for 25/70 (36%) and 8/70 (11%) regimens, respectively. Pack-regimen matching was better for WHO Watch (optimal: 15/24, 63%) than Access antibiotics (optimal: 7/39, 18%). For the four paediatric weight-examples and 42 regimens involving child-appropriate formulations, optimal and adequate packs were available for only 14/168 (8%) and 27/168 (16%), respectively. Matching was better for older children with higher body and for longer treatment courses > 7 days.

**Conclusions:**

Fixed antibiotic packs often do not match recommended treatment regimens, especially for children, potentially resulting in longer than necessary treatments and leftover doses in the community. As part of national stewardship, a move to an exact pill-count system, including for child-appropriate solid formulations, should be considered.

## Background

Antibiotic resistance is increasingly becoming a global challenge to human health [1]. The use of leftover prescribed antibiotics has been shown to be a source of self-medication and overuse of antibiotics, potentially driving resistance emergence and spread [2–4]. In Switzerland and many other countries, antibiotics are dispensed in fixed packs. Such dispensation practices appear to be a strong factor in determining possession of leftovers in the ambulatory setting [5]. A potential mismatch between clinical practice guidelines and packs could considerably influence the amount of leftover antibiotics, and therefore contribute to antibiotic misuse and overuse [6–9]. The trend towards shorter durations of antibiotic treatments could aggravate this problem if pack size remains unrevised especially for older antibiotics [10].

In 2017, the World Health Organisation (WHO) defined antibiotic groups (Access, Watch, Reserve) that are to be used globally for national stewardship based on their likely ecologic impact on antimicrobial resistance [11]. In 2019, a recommendation for at least 60% of all antibiotics used at a national level to be from the so-called Access group followed [12]. It is unclear whether there could be a differential pack-regimen mismatch for Access compared with other antibiotics, given that many Access antibiotics are older agents that came to market when longer treatment durations were favoured [10, 13]. This could potentially drive preferential prescribing of Watch antibiotics to support currently recommended shorter treatment durations.

We investigated whether antibiotic packs available in Switzerland correspond to, and therefore support compliance with, treatment regimens specified in clinical practice guidelines for common infections in children and adults treated in primary care. As a secondary objective we evaluated the types of antibiotics most commonly involved in mismatches with respect to the new WHO Access, Watch, Reserve (AWaRe) grouping of antibiotics for stewardship.

## Methods

In Switzerland, a system of compulsory health insurance covers most healthcare costs. Non-profit insurers, approved by the government, offer a basic mandatory insurance and voluntary top-up plans covering special conditions or additional services [14]. Prescribed medicines from the “list of pharmaceutical specialties”, such as oral antibiotics, are generally reimbursed by basic insurance [15, 16].

### Guidelines

Five infections frequently treated with oral antibiotics in the primary care setting in children and adults (otitis media, sinusitis, tonsillopharyngitis, community-acquired pneumonia (CAP), afebrile urinary tract infections (UTI)) were selected for this study. For these, we collected hospital-based treatment guidelines in an iterative process during July 2017–November 2017 by (i) posting a request on the online forum of the Swiss association of Public Health Administration and Hospital Pharmacists, (ii) directly contacting infectious diseases specialists at selected centres and (iii) searching online for open access hospital guidelines.

We specifically focused on obtaining information from designated training hospitals for infectious diseases. Additional guidelines from non-training hospitals were also considered and included if their recommendations deviated. When national recommendations became available in 2019 for otitis media, sinusitis, tonsillopharyngitis and adult afebrile UTI, these were included [17]. We assume that a considerable proportion of prescribers is likely to be using local older recommendations during a transitional period as well as for CAP pending national guidance.

### Antibiotic formulations and packs

All formulations, packs and brands for the antibiotics recommended in the identified guidelines were retrieved from Compendium®, the pharmaceutical register of all medications available in Switzerland, excluding those imported from foreign countries [18].

We divided oral formulations into 3 groups: solid (tablets, capsules), dispersible (tablets, granules) and liquid (suspension, powder for oral suspension). Only liquids were considered child-appropriate, because children younger than school age are unlikely to be able to swallow monolithic solid formulations [19]. Furthermore, solids and dispersible tablets are licensed only in patients weighing more than 40 kg in Switzerland, so their use would be considered off-label in young children. Slow release formulations were not included because of their absence of recommendation in the guidelines, with the exception of nitrofurantoin.

### Identification of recommended regimens

Data on the following aspects of each regimen and infection were entered onto an online REDCap™ database: recommended drug, single dose, dosing frequency and duration of treatment, recommended first or second-line or in special instances (drug allergy) [20]. Recommendations for “simple urinary tract infection” were interpreted as only applicable to women.

We analysed all possible oral antibiotic regimens for all recommended active substances by infection: For adult patients, we calculated the required number of solid or dispersible dosing units per regimen. In cases of treatment duration ranges, the required number of dosing units for the minimal and maximal duration was determined and only the extremes were evaluated as no assumptions about the commonly selected durations were possible.

As paediatric prescribing in Switzerland is weight-based, we used four weight-examples to evaluate pack and regimen matches at body weights of 10 kg (corresponding to roughly 15 months of age), 15 kg (roughly 3.5 years), 20 kg (roughly 6 years) and 25 kg (roughly 8 years) [21].

For formulations with amoxicillin and enzyme inhibitor, all calculations were based on the amoxicillin content whereas for cotrimoxazole, we considered the total amount of both substances together. For paediatric amoxicillin/clavulanate use, we limited ourselves to 7:1 ratio formulations.

### Determination of matching of available packs and regimens

Based on the above data, regimens were matched with available packs and were categorized into three groups: optimal match (no leftover doses for recommended regimen), adequate match (optimal +/− one single dose compared to recommended regimen), no match (+/− more than two doses compared to recommended regimen necessary to match pack). For children, we followed the same approach by calculating the total amount required for a treatment course for all identified regimens by weight-example. Pack-regimen matching was done as for adults but limited to child-appropriate liquid formulations. We assumed that dispensation of several packs, when required, would involve only one type of pack and formulation concentration. Combinations of different packs per drug were not considered.

### Matching patterns according to access and Watch WHO antibiotic groups

We reviewed pack-regimen matching by Access and Watch groups as defined in the 2017 revision of the WHO Model list of Essential Medicines, considering only core Access antibiotics as part of the Access group [11]. Access-Watch antibiotics were included in the Watch group. Oral fosfomycin and cefuroxime were not listed and thus excluded from this analysis.

## Results

### Guidelines

For adults, 16 different hospitals provided guidelines, of which 14 were included in our study since they provided specific recommendations for the infections of interest (Additional file 1). Together with national guidelines, there were 11 different treatment guidelines for each infection with the exception of CAP with 10 guidelines and UTI in men with only six guidelines. For children, six hospitals (three university level and three regional hospitals) provided guidelines, all were included in our study. We were not able to obtain paediatric guidelines from the French part of Switzerland. All included guidelines contained recommendations for otitis media and tonsillopharyngitis. As updated national paediatric guidelines for UTI and CAP are not yet available, there were only six guidelines for these two infections, and treatment recommendations for sinusitis were only found in five guidelines.

### Treatment regimens

For adults, 70 regimens involving 15 antibiotics differing in single dose, dosing frequency and duration were identified; some were only recommended as second-line treatment, for example in the context of penicillin allergy (Additional file 2). For children, 52 oral treatment regimens for 11 substances were identified (Additional file 2).

In general, we observed a big difference in the diversity of the recommendations between infections. In both adults and children, the greatest number of treatment regimens was identified for CAP (31 for adults, involving 7 different antibiotics with treatment durations of five to 10 days; 22 for children, involving 6 antibiotics with durations of three to 10 days). The number of regimens for sinusitis, acute otitis media and streptococcal tonsillopharyngitis was generally higher for adults than for children (32 versus 8, 28 versus 11 and 21 versus 13, respectively). As similar numbers of individual agents were involved, these differences generally were related to higher variability in combinations of single dose, dosing frequency and duration. In contrast, we found only nine different regimens for UTI in women and seven regimens for UTI in men. For afebrile urinary tract infection in children there was a bigger variety of 13 different regimens involving five antibiotics. Treatment durations for afebrile UTI were relatively short, ranging from one to 7 days depending on age, sex and antibiotic used.

### Available oral antibiotic formulations

In total, fifteen antibiotics from the above regimens were of interest with solid and at least one liquid formulation being available for nine of these (no liquid formulations for norfloxacin, levofloxacin, moxifloxacin, doxycycline, fosfomycin and nitrofurantoin, Additional file 3). Correspondingly, paediatric guidelines only made recommendations for nitrofurantoin and doxycycline in children older than 8 years not covered by our weight-examples.

### Matching of recommended regimens and packs

For adults, optimal packs were available for only 36% (25/70) of recommended regimens and for an additional 11% (8/70) packs were adequate (Table 1 and Additional file 2). For the remaining 53% (37/70), no matching packs could be found.

**Table 1:** Pack-antibiotic regimen matching (optimal and adequate) for adults

Regimen				CAP	Otitis media	Tonsillopharyngitis	Sinusitis	UTI women	UTI men
Substance	Single dose	N doses/day	Duration (in days)						
**WHO group: ACCESS**									
Amoxicillin	375 mg	3	5			15 (+ 1)			
	500 mg	3	7				21 (−1)		
	750 mg	3	7	21 (−1)	21 (− 1)		21 (− 1)		
	**1000 mg**	**2**	**5**	**10**			**10**^**a**^		
	**1000 mg**	**2**	**7**	**14**			**14**^**a**^		
	1000 mg	3	5	15 (−1)	15 (−1)^**a**^		15 (−1)^**a**^		
	1000 mg	3	7	21 (−1)			21 (− 1)		
Amoxicillin/clavulanate	625 mg	3	3					9 (+ 1)^**a**^	
	625 mg	3	7	21 (−1)	21 (−1)		21 (− 1)		
	**1000 mg**	**2**	**10**	**20**	**20**		**20**		
	**1000 mg**	**4**	**5**				**20**^**a**^		
Doxycycline	**100 mg**	**2**	**5**	**10**	**10**		**10**^**a**^		
Cotrimoxazole	**960 mg**	**2**	**5**		**10**^**a**^		**10**		**10**
	**960 mg**	**2**	**10**		**20**				
**WHO group: WATCH**									
Azithromycin	**500 mg**	**1**	**3**		**3**	**3**	**3**		
	500 mg	1	5		5(+ 1)	5(+ 1)			
Ciprofloxacin	**500 mg**	**2**	**5**						**10**
Clarithromycin	**250 mg**	**2**	**7**		**14**		**14**		
	**250 mg**	**2**	**10**			**20**			
	**500 mg**	**2**	**7**	**14**	**14**				
	**500 mg**	**2**	**10**	**20**	**20**	**20**			
Levofloxacin	**500 mg**	**1**	**5**	**5**			**5**		
	**500 mg**	**1**	**7**	**7**			**7**		
	**500 mg**	**1**	**10**				**10**		
	**500 mg**	**2**	**5**	**10**					
	**500 mg**	**2**	**7**	**14**					
Moxifloxacin	**400 mg**	**1**	**5**	**5**					
	**400 mg**	**1**	**7**	**7**			**7**		
	**400 mg**	**1**	**10**	**10**			**10**		
Norfloxacin	**400 mg**	**2**	**3**					**6**^**a**^	
**WHO group: UNCLASSIFIED**									
Cefuroxime	**250 mg**	**2**	**7**		**14**		**14**		
	**500 mg**	**2**	**7**	**14**	**14**		**14**^**a**^	**14**^**a**^	
Fosfomycin	**3000 mg**	**1**	**1**					**1**^**a**^	

^a^part of the SSI-Guidelines (ssi.guidelines.ch)

+1 = optimized by adding a single dose; -1 = optimized by subtracting a single dose

*CAP* Community-acquired pneumonia, *UTI* Urinary tract infection

The regimens are shown together with the number of dosing units required for that regimen. Antibiotics are listed alphabetically by WHO Access or Watch groups with unclassified antibiotics listed separately. Bold font indicates availability of an optimal pack for the listed antibiotic and regimen. Normal font indicates availability of an adequate pack for the listed antibiotic and regimen, meaning that matching could be achieved by adding or dropping a single dosing unit. Listed regimens represent 47.1% (33/70 regimens) for adults. All remaining regimens did not have matching optimal or adequate packs

Antibiotics with optimal matching for all recommendations were oral fosfomycin, moxifloxacin and norfloxacin. Antibiotics without matching for any recommendations were clindamycin, nitrofurantoin and phenoxymethylpenicillin. For nine of 15 antibiotics < 50% optimal or adequate pack sizes existed for the recommended regimens. Amoxicillin packs matched only a small number of regimens (2/15 regimens, 13%) as did those for amoxicillin/clavulanate (2/12 regimens, 17%), despite these two antibiotics having the highest diversity of regimens.

For children, we matched 42 treatment regimens of nine antibiotics with child-appropriate liquid formulations available for four weight-examples. Optimal packs existed only for 8% (14/168) of regimens, with adequate packs available for another 16% (27/168) (Tables 2, 3, 4, 5 and Additional file 2).

**Table 2:** Pack-antibiotic regimen matching (optimal and adequate) in children weighing 10 kg (≈15 months of age)

Regimen				CAP	Otitis media	Tonsillopharyngitis	Sinusitis	Afebrile UTI
Substance	Single dose	N doses/day	Duration (in days)					
**WHO group: ACCESS**								
Amoxicillin	**25 mg/kg**	**2**	**10**	**1**	**1**^**a**^		**1**^**a**^	
Amoxicillin/clavulanate	25 mg/kg	2	5					0.89 (+ 1)
	**26.7 mg/kg**	**3**	**7**	**1**				
	40 mg/kg	2	3					0.86 (+ 1)
	**40 mg/kg**	**2**	**7**	**1**				
	**40 mg/kg**	**2**	**14**				**1**^**a**^	
Clindamycin	7 mg/kg	3	6			1.05 (−1)^**a**^		
**WHO group: WATCH**								
Azithromycin	10 mg/kg	1	5			0.83 (+ 1)		
**WHO group: UNCLASSIFIED**								
Cefuroxime	15 mg/kg	2	5	0.86 (+ 1)	0.86 (+ 1)^**a**^	0.86 (+ 1)		0.86 (+ 1)
	15 mg/kg	2	6			1.03 (−1)^**a**^		

^a^part of the SSI-Guidelines (ssi.guidelines.ch)

+1 = optimized by adding a single dose; -1 = optimized by subtracting a single dose

*CAP* Community-acquired pneumonia, *UTI* Urinary tract infection

The regimens are shown together with the number of dosing units required for that regimen. Antibiotics are listed alphabetically by WHO Access or Watch groups with unclassified antibiotics listed separately. Bold font indicates availability of an optimal pack for the listed antibiotic and regimen. Normal font indicates availability of an adequate pack for the listed antibiotic and regimen, meaning that matching could be achieved by adding or dropping a single dosing unit to the regimen or leftovers of < one single dosing unit. Listed regimens represent 24.4% (10/42 regimens) in this weight-example. All remaining regimens did not have matching optimal or adequate packs

**Table 3:** Pack-antibiotic regimen matching (optimal and adequate) in children weighing 15 kg (≈3.5 years of age)

Regimen				CAP	Otitis media	Tonsillopharyngitis	Sinusitis	Afebrile UTI
Substance	Single dose	N doses/day	Duration (in days)					
**WHO group: ACCESS**								
Amoxicillin	25 mg/kg	2	7			1.05 (−1)		
Amoxicillin/clavulanate	25 mg/kg	2	3					0.80 (+ 1)
	25 mg/kg	2	7	0.94				
**WHO group: WATCH**								
Azithromycin	10 mg/kg	1	3	0.75 (+ 1)	0.75 (+ 1)			
	10 mg/kg	1	5			1.25 (−1)		
**WHO group: UNCLASSIFIED**								
Cefuroxime	15 mg/kg	2	7	1.8 (+ 1)				

+ 1 = optimized by adding a single dose; − 1 = optimized by subtracting a single dose

*CAP* Community-acquired pneumonia, *UTI* Urinary tract infection

The regimens are shown together with the number of dosing units required for that regimen. Antibiotics are listed alphabetically by WHO Access or Watch groups with unclassified antibiotics listed separately. Bold font indicates availability of an optimal pack for the listed antibiotic and regimen. Normal font indicates availability of an adequate pack for the listed antibiotic and regimen, meaning that matching could be achieved by adding or dropping a single dosing unit to the regimen or leftovers of < one single dosing unit. Listed regimens represent 14.6% (6/42 regimens) in this weight-example. All remaining regimens did not have matching optimal or adequate packs

**Table 4:** Pack-antibiotic regimen matching (optimal and adequate) in children weighing 20 kg (≈6 years of age)

Regimen				CAP	Otitis media	Tonsillopharyngitis	Sinusitis	Afebrile UTI
Substance	Single dose	N doses/day	Duration (in days)					
**WHO group: ACCESS**								
Amoxicillin	**25 mg/kg**	**2**	**5**		**1**^**a**^			
	**25 mg/kg**	**2**	**10**	**2**	**2**^**a**^		**2**^**a**^	
Amoxicillin/clavulanate	25 mg/kg	2	5					0.89 (+ 1)
	26.7 mg/kg	3	3					0.86 (+ 1)
	**26.7 mg/kg**	**3**	**7**	**1**				
	40 mg/kg	2	3					0.86 (+ 1)
	**40 mg/kg**	**2**	**7**	**1**				
	**40 mg/kg**	**2**	**14**				**2**^**a**^	
Clindamycin	7 mg/kg	3	6			2.1 (−1)^**a**^		
	**10 mg/kg**	**3**	**10**			**5**		
**WHO group: WATCH**								
Azithromycin	**10 mg/kg**	**1**	**3**	**1**	**1**			
	10 mg/kg	1	5			0.83 (+ 1)		
**WHO group: UNCLASSIFIED**								
Cefuroxime	15 mg/kg	2	5	1.7 (+ 1)	1.7 (+ 1)^**a**^	1.7 (+ 1)		1.7 (+ 1)
	15 mg/kg	2	6			2.06 (−1)^**a**^		
	15 mg/kg	2	14				4.8 (+ 1)^**a**^	

^a^part of the SSI-Guidelines (ssi.guidelines.ch)

+ 1 = optimized by adding a single dose; − 1 = optimized by subtracting a single dose

*CAP* Community-acquired pneumonia, *UTI* Urinary tract infection

The regimens are shown together with the number of dosing units required for that regimen. Antibiotics are listed alphabetically by WHO Access or Watch groups with unclassified antibiotics listed separately. Bold font indicates availability of an optimal pack for the listed antibiotic and regimen. Normal font indicates availability of an adequate pack for the listed antibiotic and regimen, meaning that matching could be achieved by adding or dropping a single dosing unit to the regimen or leftovers of < one single dosing unit. Listed regimens represent 36.6% (15/42 regimens) in this weight-example. All remaining regimens did not have matching optimal or adequate packs

**Table 5:** Pack-antibiotic regimen matching in children (optimal and adequate) weighing 25 kg (≈8 years of age)

Regimen				CAP	Otitis media	Tonsillopharyngitis	Sinusitis	Afebrile UTI
Substance	Single dose	N doses/day	Duration (in days)					
**WHO group: ACCESS**								
Amoxicillin	**40 mg/kg**	**2**	**5**	**2**				
	40 mg/kg	2	7	2.8 (+ 1)				
Amoxicillin/clavulanate	26.7 mg/kg	3	5					0.89 (+ 1)
	40 mg/kg	2	5	0.89 (+ 1)	0.89 (+ 1)^**a**^			0.89 (+ 1)
Cotrimoxazole	18 mg/kg	2	5		0.94			0.94
	18 mg/kg	2	10		1.88 (+ 1)			
**WHO group: WATCH**								
Azithromycin	10 mg/kg	1	3	1.25 (−1)	1.25 (− 1)			
	10 mg/kg	1	5			1.04 (−1)		
**WHO group: UNCLASSIFIED**								
Cefuroxime	**15 mg/kg**	**2**	**7**	**3**				
	**15 mg/kg**	**2**	**14**				**6**^**a**^	

^a^part of the SSI-Guidelines (ssi.guidelines.ch)

+ 1 = optimized by adding a single dose; − 1 = optimized by subtracting a single dose

*CAP* Community-acquired pneumonia, *UTI* Urinary tract infection

The regimens are shown together with the number of dosing units required for that regimen. Antibiotics are listed alphabetically by WHO Access or Watch groups with unclassified antibiotics listed separately. Bold font indicates availability of an optimal pack for the listed antibiotic and regimen. Normal font indicates availability of an adequate pack for the listed antibiotic and regimen, meaning that matching could be achieved by adding or dropping a single dosing unit to the regimen or leftovers of < one single dosing unit. Listed regimens represent 24.4% (10/42 regimens) in this weight-example. All remaining regimens did not have matching optimal or adequate packs

For the remaining 76% (127/168), no matching packs could be found. We observed best matching for amoxicillin regimens (4/28, 14%). Despite three different available pack sizes for amoxicillin/clavulanate and 13 different regimens, only 12% (6/52) had optimal packs. We could not find any suitable packs for the considered weight-examples for ciprofloxacin, clarithromycin, phenoxymethylpenicillin and cotrimoxazole. For lighter and therefore younger children (10 kg, 15 kg) matching was poorer than for heavier older children (20 kg, 25 kg). Comparing optimal versus adequate packs, there were more optimal packs available for regimens with longer duration, especially in younger children.

### Matching according to the WHO AWaRe groups

For adults, optimal and adequate packs existed for only 18% (7/39) and 18% (7/39) regimens involving Access antibiotics (Table 1 and Additional file 4). For the remaining 64% (25/39) no matching packs could be found. In contrast optimal packs were available for 63% (15/24) of regimens involving Watch antibiotics.

For children, a very low rate of optimal available packs was found for both groups (9% in the Access group, 4% in the Watch group, Tables 2, 3, 4, 5 and Additional file 4). We also observed a larger range of different regimens (*N* = 12–52) for Access antibiotics than for Watch antibiotics (*N* = 4–12).

## Discussion

Our analysis of pack-regimen matching showed an important mismatch between recommended oral regimens for infections commonly treated in the community setting and available pack sizes for antibiotics in Switzerland.

For adults, a suitable pack could be found for only 36% of the considered regimens, and for child-appropriate formulations this rate was even lower at 8% based on four weight-examples. In adults, all antibiotics with high rates of suitable packs were from the WHO Watch group [10]. This is an alarming fact from the perspective of antibiotic stewardship: Dispensing of Watch antibiotics in Switzerland seems paradoxically easier than dispensing of Access antibiotics, with better pack-regimen matching.

For children very low rates of suitable packs were available. Moving from exact weight-based to weight-banded dosing with appropriate packs, and greater advocacy for the development and licensing of dispersible or mini-tablets could mitigate some of the specific challenges [19, 22]. Indeed, despite the current situation, use of child-appropriate formulations of Access antibiotics in Switzerland is high (> 85%) [23]. Packs often seem adapted to cater to older (and heavier) children and longer treatment durations with poorer compliance [24]. Considering they experience the highest rates of community-based antibiotic prescribing, the fewest suitable packs for evidence-based shorter treatment durations were available for children less than 5 years of age [23]. In contrast, some regimens could only be achieved by dispensing multiple packs, increasing the risk that a second package is not administered and kept at home for future use as well as of environmental contamination on disposal.

Similar mismatches between recommended regimens and available pack sizes have been documented in Croatia, India and Australia, with this phenomenon thought to account for a substantial proportion of “redundant antibiotic doses in the community” [6, 8, 9]. Antibiotics dispensed in fixed packs seem to be a risk factor for leftovers in adults. Kardas and colleagues describe a higher tendency for patients in “pack-countries” to save leftovers or give them to others than in “pill-countries” where the exact amount of pills required per course is dispensed [5]. Leftover antibiotics are also often disposed in the household waste or flushed down the toilet which can contribute to the development of antibiotic resistance [25].

The strength of our study is that we most likely were able to identify the majority of frequently used regimens for five infections commonly managed in primary care and combined these with comprehensive information on available antibiotic packs in Switzerland. Furthermore, we covered the whole population of adults and children in our analysis. We anticipate that our observations will be applicable to many other pack-dispensing countries.

Our study also has limitations. We focused on hospital guidelines and very recent national recommendations. In general, all community prescribers in Switzerland will undergo hospital-based training, and current prescribers will most likely have been exposed to hospital antibiotic policies during their training [26, 27]. We also selected certain infections based on their considerable contribution to antibiotic prescribing in the community. Antibiotics and treatment regimens may differ for other common infections, such as skin and soft tissue infections, with potentially better matching with available packs. When treatment ranges were given, we only considered the minimal and maximal duration, but durations in between may also be chosen. In certain cases, such an intermediate duration could provide an optimal or adequate match with an available pack. Of note, some intermediate durations may actually have been represented in another included recommendation. We incorporated the regimens with one missing single dose in the adequate match group because we considered one missing dose of a regimen to be clinically acceptable. However, this could be result in the dispensation of an additional full pack and increase the risk of leftover antibiotics. In children, we analysed only four weight examples and sometimes slightly higher or lower weight may achieve good matching. This makes an exact evaluation complex, as leftover dose units will differ by weight. It also demonstrates the potential negative impact of exact weight-based dosing using suspensions on matching of treatment recommendations and available pack sizes. Finally, even with perfect matching of available packs and treatment regimens adherence may be suboptimal and doses may be left over.

## Conclusions

This study highlights that fixed packs of antibiotics in Switzerland often do not fit with the recommended treatment regimens. Influencing pack sizes to ensure that optimal packs for shortest evidence-based regimens are available is challenging, with complex economic and regulatory implications. Furthermore, the same antibiotics are likely to be used differently for the treatment of different infections. As part of national stewardship efforts, consideration should therefore be given to moving to an exact pill-count system for adults and advocating for weight-banded dosing approaches or child-appropriate solid formulations for children. Exact pill-count dispensation in particular would support wider use of short Access antibiotic treatment courses and could enable personalised treatment regimens based on stratification factors in the future.

## Supplementary information

**Additional file 1.** Sources of Guidelines. Source hospitals providing guidelines used as basis for the study.

**Additional file 2.** Guidelines and pack size matching. Availability of optimal, adequate and no matching pack sizes for all relevant antibiotics but not specific to indication. Data are shown separately for adults (Table 2a) and children (Table 2b).

**Additional file 3.** Available formulations in Switzerland for guideline-relevant antibiotics. Available formulations shown by beta-lactams (Table 3a), quinolones (Table 3b) and other antibiotics (Table 3c); both solid and liquid formulations were included.

**Additional file 4.** Guidelines and pack size matching according to the WHO AWaRe groups. Data are shown separately for adults (Table 4a) and children (Table 4b).

## Data Availability

The datasets used and/or analysed during the current study are available from the corresponding author on reasonable request.

## Abbreviations

AWaRe: Access, Watch, Reserve
CAP: Community-acquired pneumonia
UTI: Urinary tract infections
WHO: World Health Organisation
