# Supplementary material for: The potential negative impact of antibiotic pack on antibiotic stewardship in primary care in Switzerland: a modelling study
Source: Antimicrob Resist Infect Control. 2020 May 8;9:60. doi: 10.1186/s13756-020-00724-7 (PMC7206713; doi:10.1186/s13756-020-00724-7)
Supplement: Supplementary file 1 — Additional file 1. Sources of Guidelines. Source hospitals providing guidelines used as basis for the study. [file 13756_2020_724_MOESM1_ESM.pdf]

Additional file 1: Sources of Guidelines

Table 1a: Included references of the guidelines for adults

Source hospital	CAP	Tonsillo-pharyngitis	Otitis media	Sinusitis	Afebrile UTI	
					Women	Men
Basel*	✓	X	X	X	✓	✓
Bern*	✓	✓	✓	✓	✓	✓
Zürich*	✓	✓	✓	✓	✓	X
Aarau	✓	✓	✓	✓	✓	✓
Luzern	✓	✓	✓	✓	✓	X
Genf [1*]	✓	✓	✓	✓	✓	✓
St. Gallen	✓	✓	✓	✓	✓	✓
Wallis	✓	✓	✓	✓	✓	X
Lausanne*	✓	✓	✓	✓	✓	✓
Tessin	✓	✓	✓	✓	✓	✓
National Guidelines	X	✓	✓	✓	✓	X

[1] 3 different references from the same hospital; *University hospital

Table 1b: Included references of the guidelines for children

Source hospital	CAP	Tonsillopharyngitis	Otitis media	Sinusitis	Afebrile UTI
Basel*	✓	✓	✓	X	✓
Zürich*	✓	✓	✓	X	✓
St. Gallen	✓	✓	✓	✓	✓
Bern*	✓	✓	✓	✓	✓
Luzern	✓	✓	✓	✓	✓
Tessin	✓	✓	✓	✓	✓
National Guidelines	X	✓	✓	✓	X

*University hospital
