# Supplementary material for: The potential negative impact of antibiotic pack on antibiotic stewardship in primary care in Switzerland: a modelling study
Source: Antimicrob Resist Infect Control. 2020 May 8;9:60. doi: 10.1186/s13756-020-00724-7 (PMC7206713; doi:10.1186/s13756-020-00724-7)
Supplement: Supplementary file 2 — Additional file 2. Guidelines and pack size matching. Availability of optimal, adequate and no matching pack sizes for all relevant antibiotics but not specific to indication. Data are shown separately for adults (Table 2a) and children (Table 2b). [file 13756_2020_724_MOESM2_ESM.pdf]

Additional file 2: Guidelines and pack size matching

Table 2a: Matching of recommended regimens and packs for adults

Substance	N possible different single dose/frequency /duration combinations	correct available pack size	single dose +/-	no available pack size
Amoxicillin	15	2 (13.3%)	5 (33.3%)	8 (53.3%)
Amoxicillin/clavulanate	12	2 (16.7%)	2 (16.7%)	8 (66.7%)
Azithromycin	3	1 (33.3%)	1 (33.3%)	1 (33.3%)
Cefuroxime	6	2 (33.3%)	0 (0%)	4 (66.7%)
Ciprofloxacin	3	1 (33.3%)	0 (0%)	2 (66.7%)
Clarithromycin	7	4 (57.1%)	0 (0%)	3 (42.9%)
Clindamycin	2	0 (0%)	0 (0%)	2 (100%)
Cotrimoxazole	4	2 (50%)	0 (0%)	2 (50%)
Doxycyclin	2	1 (50%)	0 (0%)	1 (50%)
Fosfomycin	1	1 (100%)	0 (0%)	0 (0%)
Levofloxacin	7	5 (71.4%)	0 (0%)	2 (28.6%)
Moxifloxacin	3	3 (100%)	0 (0%)	0 (0%)
Nitrofurantoin	2	0 (0%)	0 (0%)	2 (100%)
Norfloxacin	1	1 (100%)	0 (0%)	0 (0%)
Phenoxymethylpenicillin	2	0 (0%)	0 (0%)	2 (100%)
Total 15 different substances	Total 70 different regimens	25 (35.7%)	8 (11.4%)	37 (52.9%)

Table 2b: Matching of recommended regimens and packs for children. The regimens are calculated for four weight-examples.

Substance	N possible different single dose/frequency /duration combinations	correct available pack size	single dose +/-	no available pack size
Amoxicillin	28	4 (14.3%)	2 (7.1%)	22 (78.6%)
Amoxicillin/clavulanate (7:1)	52	6 (11.5%)	9 (17.3%)	37 (71.2%)
Azithromycin	8	1 (12.5%)	6 (75%)	1 (12.5%)
Cefuroxime	20	2 (10%)	6 (30%)	12 (60%)
Ciprofloxacin	4	0 (0%)	0 (0%)	4(100%)
Clarithromycin	12	0 (0%)	0 (0%)	12 (100%)
Clindamycin	12	1 (8.3%)	2 (16.7%)	9 (75%)
Cotrimoxazole	16	0 (0%)	2 (12.5%)	14 (87.5%)
Phenoxymethylpenicillin	16	0 (0%)	0 (0%)	16 (100%)

Total 9 different substances	Total 168 different regimens	14 (8.3%)	27 (16.1%)	127 (75.6%)
