# Supplementary material for: The potential negative impact of antibiotic pack on antibiotic stewardship in primary care in Switzerland: a modelling study
Source: Antimicrob Resist Infect Control. 2020 May 8;9:60. doi: 10.1186/s13756-020-00724-7 (PMC7206713; doi:10.1186/s13756-020-00724-7)
Supplement: Supplementary file 3 — Additional file 3. Available formulations in Switzerland for guideline-relevant antibiotics. Available formulations shown by beta-lactams (Table 3a), quinolones (Table 3b) and other antibiotics (Table 3c); both solid and liquid formulations were included. [file 13756_2020_724_MOESM3_ESM.pdf]

Additional file 3: Available formulations in Switzerland for guideline-relevant antibiotics

Table 3a: Beta-lactams

	**Concentration**	**Pack size (number of dosage)**	**No. of brands**
**AMOXICILLIN**			
Solid	375 mg	16	2
	500 mg	20	2
	750 mg	20	2
	1000 mg	14/20	2
Dispersible	750 mg	4/20	1
	1000 mg	3/10/20	1
Liquid	250 mg/5 ml	20 (5000 mg)	2
**AMOXICILLIN/ CLAVULANATE**			
Solid	250 mg:125 mg (375 mg)	16	1
	500 mg:125 mg (625 mg)	10/20	5
	875 mg:125 mg (1000 mg)	12/20	5
Dispersible	500 mg:125 mg (625 mg)	10/20	2
	875 mg:125 mg (1000 mg)	12/20	2
Liquid	125 mg:31.25 mg/5ml	20 (2500 mg)	2
	250 mg:62.5 mg/5ml	20 (5000 mg)	4
	400 mg:57 mg /5ml	7 (2800 mg)/14 (5600 mg) /28 (11200 mg)	4
**PHENOXYMETHYLPENICILLIN (PENICILLIN V)**			
Solid	1 MIO IU	12/24	1
	1.5 MIO IU	12/24	1
Dispersible	-		
Liquid	400‘000 IU/5 ml	12 (4.8 Mio IU)/24 (9.6 Mio IU)	1
	750‘000 IU/5 ml	12 (9 Mio IU)/24 (18 Mio IU)	1
**CEFUROXIME**			
Solid	250 mg	14	5
	500 mg	14	5
Dispersible	-		
Liquid	125 mg/5 ml	14 (1750 mg)	1

Table 3b: Quinolones

**NORFLOXACIN**			
Solid	400 mg	6/7/14/42	3
Dispersible	-		
Liquid	-		
**CIPROFLOXACIN**			
Solid	250 mg	6/10/20	9
	500 mg	10/20	9
	750 mg	20	9
Dispersible	-		
Liquid	250 mg/5 ml	20 (5000 mg)	1
	500 mg/5 ml	20 (10000 mg)	1
**LEVOFLOXACIN**			
Solid	250 mg	5/7/10	5

	500 mg	5/7/10	5
Dispersible	-		
Liquid	-		
**MOXIFLOXACIN**			
Solid	400 mg	5/7/10	4
Dispersible/Liquid	-		

Table 3c: Other antibiotics

**AZITHROMYCIN**			
Solid	250 mg	4/6	5
	500 mg	3	4
Dispersible	100 mg	3	1
	300 mg	3	1
	500 mg	3	1
Liquid	200 mg/5 ml	3 (600 mg)/6 (1200 mg)	4
**CLARITHROMYCIN**			
Solid	250 mg	14/20	4
	500 mg	14/20/30	4
	750 mg	30	1
Dispersible	-		
Liquid	125 mg/5 ml	20 (2500 mg)	4
	250 mg/5 ml	20 (5000 mg)	4
**CLINDAMYCIN**			
Solid	150 mg	16	4
	300 mg	16	4
Dispersible	-		
Liquid	75 mg/5 ml	16 (1200 mg)	2
**COTRIMOXAZOLE**			
Solid	960 mg	10/20/50	2
Dispersible	-		
Liquid	240 mg/5 ml	20 (4800 mg)	1
**DOXYCYCLINE**			
Solid	100 mg	8/10/20/32	5
	200 mg	8/10	5
Dispersible	-		
Liquid	-		
**FOSFOMYCIN**			
Solid	-		
Dispersible	2000 mg	1	1
	3000 mg	1	2
Liquid	-		
**NITROFURANTOIN**			
Solid	100 mg	20/30/50	2
Dispersible	-		
Liquid	-		
