# Supplementary material for: The potential negative impact of antibiotic pack on antibiotic stewardship in primary care in Switzerland: a modelling study
Source: Antimicrob Resist Infect Control. 2020 May 8;9:60. doi: 10.1186/s13756-020-00724-7 (PMC7206713; doi:10.1186/s13756-020-00724-7)
Supplement: Supplementary file 4 — Additional file 4. Guidelines and pack size matching according to the WHO AWaRe groups. Data are shown separately for adults (Table 4a) and children (Table 4b). [file 13756_2020_724_MOESM4_ESM.pdf]

Additional file 4: Matching according to the WHO AWaRe groups

Table 4a: Pack size-regimen matching according to the WHO AWaRe groups for adults.

	N possible different single dose/frequency /duration combinations	correct available pack size	single dose +/-	no available pack size
**Access**				
Amoxicillin	15	2 (13.3%)	5 (33.3%)	8 (53.3%)
Amoxicillin/ clavulanate	12	2 (16.7%)	2 16.7(%)	8 (66.7%)
Clindamycin	2	0 (0%)	0 (0%)	2 (100%)
Cotrimoxazole	4	2 (50%)	0 (0%)	2 (50%)
Doxycycline	2	1 (50%)	0 (0%)	1 (50%)
Nitrofurantoin	2	0 (0%)	0 (0%)	2 (100%)
Phenoxymethyl-penicillin	2	0 (0%)	0 (0%)	2 (100%)
Total	39	7 (17.9%)	7 (17.9%)	25 (64.1%)
**Watch**				
Azithromycin	3	1 (33.3%)	1 (33.3%)	1 (33.3%)
Ciprofloxacin	3	1 (33.3%)	0 (0%)	2 (66.7%)
Clarithromycin	7	4 (57.1%)	0 (0%)	3 (42.9%)
Levofloxacin	7	5 (71.4%)	0 (0%)	2 (28.6%)
Moxifloxacin	3	3 (100%)	0 (0%)	0 (0%)
Norfloxacin	1	1 (100%)	0 (0%)	0 (0%)
Total	24	15 (62.5%)	1 (4.2%)	8 (33.3%)

Table 4b: Pack size-regimen matching according to the WHO AWaRe groups for children.

	N possible different single dose/frequency/ duration	correct available pack size	single dose +/-	no available pack size
**Access**				
Amoxicillin	28	4 (14.3%)	2 (7.1%)	22 (78.6%)
Amoxicillin/ clavulanate	52	6 (11.5%)	9 (17.3%)	37 (71.2%)
Clindamycin	12	1 (8.3%)	2 (16.7%)	9 (75%)
Cotrimoxazole	16	0 (0%)	2 (12.5%)	14 (87.5%)
Phenoxymethylp enicillin	16	0 (0%)	0 (0%)	16 (100%)
Total	124	11 (8.9%)	15 (12.1%)	98 (79%)
**Watch**				
Azithromycin	8	1 (12.5%)	6 (75%)	1 (12.5%)
Ciprofloxacin	4	0 (0%)	0 (0%)	4(100%)
Clarithromycin	12	0 (0%)	0 (0%)	12 (100%)
Total	24	1 (4.2%)	6 (25%)	17 (70.8%)

Cefuroxime and oral fosfomycin are not included because they were unclassified at the time of writing.
